# Conventional fractionation should not be the standard of care for T2 glottic cancer

**DOI:** 10.1186/s13014-017-0915-8

**Published:** 2017-11-14

**Authors:** Lynne M. Dixon, Catriona M. Douglas, Shazril Imran Shaukat, Kate Garcez, Lip Wai Lee, Andrew J. Sykes, David Thomson, Nicholas J. Slevin

**Affiliations:** 1 Department of Clinical Oncology, The Christie NHS Foundation Trust, Wilmslow Road, Manchester, M20 4BX UK; 2 Department of Otolaryngology – Head and Neck Surgery, Queen Elizabeth University Hospital, 1345 Govan Road, Glasgow, G51 4TF UK

**Keywords:** T2 Glottic, Biologically effective dose, Hypofractionation, Local control, Radiotherapy

## Abstract

**Background:**

The aim of this study was to report outcomes and late toxicity following hypofractionated accelerated radiotherapy for T2 glottic cancers. We highlight the importance of hypofractionated treatments with shorter overall treatment times, in improving outcomes for T2 glottic cancers. We also compare the biologically effective dose of hypofractionated regimes, with conventional fractionation.

**Methods:**

One hundred twelve patients with T2 glottic cancer were treated between January 1999 and December 2005. All patients were prescribed a hypofractionated accelerated radiotherapy dose of 52.5 Gray in 3.28 Gray per fraction, delivered over 22 days. Radiobiological calculations were used to assess the relationship of fraction size and overall treatment time on local control outcomes and late toxicity.

**Results:**

The 5-year overall survival was 67%, the 5-year local control was 82%, and the 5-year disease-specific survival was 90%. The respective 5-year local control for T2a and T2b disease was 88.8 and 70.8% (*p* = 0.032). Severe late toxicity occurred in two patients (1.8%). Radiobiological calculations showed an increase in local control of nearly 12%, with a 10 Gray increase in biologically effective dose.

**Conclusion:**

This study has demonstrated that accelerated hypofractionated regimes have improved local control and similar late toxicity compared with conventional fractionation schedules. This supports the use of hypofractionated regimes as the standard of care for early glottic laryngeal cancers.

## Background

Larynx cancer accounts for 2.4% of all cancers [1]. Early glottic larynx cancers (T1–2N0) have similar local control rates when treated with radiotherapy or surgery [2]. Due to potential for superior voice quality following treatment, the general approach would be for primary radiotherapy, with conservative surgery in reserve [3].

The 5-year local control rates for T1 glottic cancer treated with radiotherapy are 85 to 95%, with ultimate control (where salvage surgery required) between 95 and 100% [4–6]. For T2 glottic cancer treated with radiotherapy the 5-year local control rates are 65–85%, and 75–95% following surgical salvage [7, 8]. Using conventionally fractionated radiotherapy and international standard radical head and neck radiotherapy regimen of 70 Gray (Gy) delivered in 2 Gy per fraction over 7 weeks, the local control rate for T2 glottic cancer is only approximately 70% (Table 1). Reports from various alternative fractionation schedules including our presented data, suggest improved local control, with no increase in late effects [9, 10].

**Table 1:** Local control for dose regimes of 70 Gy, in 2Gy per fraction

Series	Year	No. of patients	5-year LC (%)
Berwouts [16]	2015	81	75
Trotti [17]	2014	119	70
Howell-Burke [18]	1990	114	72
Garden [7]	2003	230	68

Here we report our local control and survival outcomes and toxicity data using an accelerated hypofractionated radiotherapy schedule. Our cohort received 52.5 Gy in 3.28 Gy per fraction, with an overall treatment time of 22 days. Royal College of Radiologists guidance now recommends hypofractionated regimes for the treatment of early glottic cancers, and our current practice has changed to 55 Gy in 2.75 Gy per fraction, in line with these guidelines [11]. This review highlights the importance of hypofractionation, and shorter overall treatment times, in improving outcomes for T2 glottic cancers. We compare the biologically effective dose (BED) for hypofractionated and conventional radiotherapy regimes, and determine the dose-response gradient for T2 glottic cancers. We also discuss consideration of surgical treatment options or chemoradiotherapy for T2b tumours.

## Methods

Patients were treated between January 1999 and December 2005. The inclusion criteria were a histologically confirmed AJCC T2N0 squamous cell carcinoma of the glottic larynx, with radiotherapy as the primary treatment modality. Exclusion criteria were not completing the full course of treatment, or prior radical surgery. All patients had computed tomography (CT) scans for staging and nodal evaluation, and endoscopic assessments were used for local staging. Ultrasound scans were not routinely used. All patients were staged using TNM 7th [12].Patient records were analysed to determine patient, treatment and tumour factors.

Radiotherapy was delivered as a single phase treatment, using lateral parallel opposed or anterior oblique paired fields, as previously described [13]. Anterior oblique fields were used for small volume T2a tumours, while lateral fields were used for larger volume and T2b tumours. There was no elective nodal irradiation, however lateral fields would have coincidentally encompassed part of level 3 nodal regions at the level of the larynx. Field sizes were 5.5–7 cm in cranio-caudal length. All patients received accelerated hypofractionated radiotherapy, with a prescribed dose of 52.5 Gy in 16 once daily fractions of 3.28 Gy, Monday–Friday over 3 weeks. None of the patients in our cohort received any induction, concurrent or adjuvant chemotherapy.

The primary outcome measures were 5-year overall survival (OS), 5-year local control (LC), and 5-year disease specific survival (DSS). Secondary outcome measures were severe morbidity, defined by the Common Terminology Criteria for Adverse Events, as Grade 4 laryngeal oedema, mucositis or obstruction [14]. Outcome data was collected a minimum of 2 years following start of radiotherapy, and all times to events were calculated in days from the start of radiotherapy. Kaplan-Meier curves were used for survival, and compared using the log-rank test. The factors analysed for survival and disease free survival were age, sex, pre-treatment haemoglobin, cord mobility (T2a normal and T2b impaired mobility), smoking status and alcohol intake.

## Results

### Patient demographics

Between January 1999 and December 2005, 112 patients were treated for T2N0 glottic squamous cell carcinoma. There were 98 (88%) males and median age was 64 (range, 38–85 years). Seventy-six patients (68%) were staged as T2a, and 36 (32%) had T2b disease, as shown in Table 2.

**Table 2:** Prognostic factors for 5-year local control

Factor	Subgroup	Number (%)	5-year LC % (95% CI)	*p* value (log rank)
Sex	Male	98 (88)	84.8% (75.5–90.8)	0.20
	Female	14 (13)	70.0% (38.3–87.6)	
Age (years)	<65	58 (52)	73.2% (59.9–82.9)	0.003
	≥65	54 (48)	93.9% (82.3–98.0)	
Stage	2a	76 (68)	88.8% (79.0–94.2)	0.032
	2b	36 (32)	70.8% (52.4–83.1)	
Haemoglobin (g/dl)	<13	42 (38)	89.4% (74.2–95.9)	0.46
	≥13	60 (54)	81.1% (68.3–89.1)	
	Unknown	10 (9)		
Smoker	Current/ex <1 yr	46 (41)	79.0% (63.6–88.4)	0.42
	Ex ≥ 1 year/never	64 (57)	87.0% (75.6–93.3)	
	Unknown	2 (2)		
Alcohol	Low/no alcohol	69 (62)	82.5% (70.6–89.9)	0.41
	Heavy/previous heavy	29 (26)	89.6% (71.0–96.5)	
	Unknown	14 (13)		

Although 5-year LC in smokers was worse than that for non-smokers, this was not statistically significant (78% vs 87%, *p* = 0.42). Younger patients aged under 65 did significantly worse than older patients, with inferior 5-year LC (73% vs 94%, *p* = 0.003).

### Survival

The median follow up was 5.8 years (range 0.1–9.6 years). All but four cases were followed up for at least 3 years, or had disease recurrence before that point. Nineteen (17%) patients developed recurrence, of these 11 (9%) were within the radiotherapy field, 4 (4%) were nodal recurrence alone, and 4 (4%) were both in field and nodal recurrences. There were 18 (16%) patients who developed second malignancies in the lung, kidney or bladder.

The 5-year OS was 67%, 5-year LC was 82%, and 5-year DSS was 90%. The respective 5-year LC rate for T2a and T2b disease was 88.8 and 70.8% (*p* = 0.032). Age less than 65 years was associated with poorer outcome, 5-year LC of 73.2%, compared with 93.9% in those aged 65 or over (*p* = 0.003), as shown in Table 2.

### Post treatment morbidity

There was a low rate of severe late morbidity (defined as requiring surgical intervention); 2 (1.8%) patients who required a tracheostomy or total laryngectomy. Twelve (10.7%) patients had a total laryngectomy for disease recurrence.

## Discussion

### Key findings

For T2N0 glottic larynx cancer, a hypofractionated schedule of 52.5 Gy in 3.28 Gy per fraction, resulted in 5-year loco-regional control rate of 82%. This is comparable with other hypofractionated treatment regimens, demonstrating local control rates of 76–82% [9, 10, 15]. However, the current international standard dose fractionation for head and neck cancer of 70 Gy, in 2 Gy per fraction (Gy/#), delivered over 7 weeks is associated with a 5-year LC rate of 68–75% [7, 16–18]. This raises the question as to whether hypofractionated schedules for early glottic larynx cancer should be adopted as a new standard. Despite excellent outcomes for T2a tumours, results were more disappointing for T2b tumours, with only 71% 5-year LC, and therefore alternative treatment strategies using either surgery or chemoradiotherapy, should be considered, and are discussed later.

In our cohort there was no significant difference in local control dependent on haemoglobin levels, although this has been shown to have a prognostic impact on local control in other series [5]. A recent meta-analysis also found low pre-treatment haemoglobin to be a major influential factor for radiation failure in early glottic cancers (relative risk 0.891, *p* < 0.001) [19]. Another unexpected result was a statistically significant inferior local control in younger patients aged under 65. Generally it would be expected to see worse outcomes in an older cohort, but there was no multivariate analysis on our subgroups to interpret this result further.

Outcomes from radiotherapy for early glottic larynx cancer have improved over time [6, 8, 9], likely due to multiple factors including; improved staging accuracy with introduction of routine pre-treatment CT scanning, all patients being discussed at an multidisciplinary team meeting, and national cancer guidelines for head and neck [20, 21]. Glottic tumours with inner cortex thyroid cartilage invasion and/or cord fixation and/or invasion of the paraglottic space are staged as T3. While flexible nasendoscopy will demonstrate a fixed hemi-larynx, thyroid cartilage invasion and paraglottic space invasion are best assessed on CT. Several studies have demonstrated that endoscopic assessment frequently understages laryngeal cancer and demonstrated the importance of CT imaging in providing accurate staging information [22, 23]. As patients are now staged more accurately, due to the advances in imaging technology, this has potentially resulted in more patients being upstaged to T3 and an apparent improved outcome for T2 tumours (*Will Rogers* effect) [23].

### Radiobiology

From a radiobiological perspective, the main factors influencing local control are total dose, dose per fraction and overall treatment time. The DAHANCA6 trial compared six fractions per week with five fractions per week, using 2 Gy/#, showing a reduced risk of recurrence with accelerated regimes for T1–T2 glottic cancers (HR 0.6, CI 0.41–0.89) [24]. This study also found benefit from acceleration in well and moderately differentiated tumours, suggesting that accelerated repopulation requires cells to have adequate functional behaviour to respond to the radiation trauma, and these mechanisms are more pronounced in differentiated cells [24]. Our study was limited by lacking histological differentiation for our cohort, which may significantly affect outcomes from our accelerated fractionation regime.

To compare local control outcomes for different schedules, BED was calculated for different dose fractionation regimens, using the following equation [25]:

$$ {\displaystyle \begin{array}{l} Biologically effective dose(BED)=D\left(1+d/\alpha /\beta \right)-\left( OTT\hbox{--} L\right)\times T\\ {}\left(D= total dose,d= dose\; per\; fraction, OTT= overall treatment time,L= time\; lag,T= time factor\right)\end{array}} $$

Calculations included time lag and time factor, to account for the effect of repopulation. Accepted values for time factor range from 0.5 to 1 [26, 27], while time lag is usually considered to be between 21 and 28 days [25, 27]. Assuming fraction size to be more influential than overall treatment time, the initial value chosen for time factor was 0.6, with a time lag of 28 days. Two regimens that result in similar local control rates of 70% are 70Gy in 35# over 7 weeks, and 50Gy in 20# over 4 weeks. The BED for the tumour (BED10) was therefore calculated for these regimens, using the above equation, (using α/β of 10):

$$ {\displaystyle \begin{array}{l} BED 10\; for\; 70 Gy/ 35\#= 73 Gy\\ {} BED 10\; for\; 50 Gy/ 20\#= 63 Gy\end{array}} $$

The values for these regimens do not correlate with their similar local control rates, and show a 10Gy difference in BED. To place more emphasis on overall treatment time, the value for time lag was changed from 28 to 21 days, and BED10 recalculated:

$$ {\displaystyle \begin{array}{l}\mathrm{BED}10\;\mathrm{for}\;70\mathrm{Gy}/35\#=68\mathrm{Gy}\\ {}\mathrm{BED}10\;\mathrm{for}\;50 Gy/20\#=58\mathrm{Gy}\end{array}} $$

These values remain different by 10Gy, suggesting that time factor is more influential, hence this was increased from 0.6 to 1.0, and BED10 was recalculated using an intermediate value of 25 days for time lag.

$$ {\displaystyle \begin{array}{c} BED 10\; for\; 70 Gy/ 35\#= 62 Gy\\ {} BED 10\; for\; 50 Gy/ 20\#= 60 Gy\end{array}} $$

**Fig. 1:**
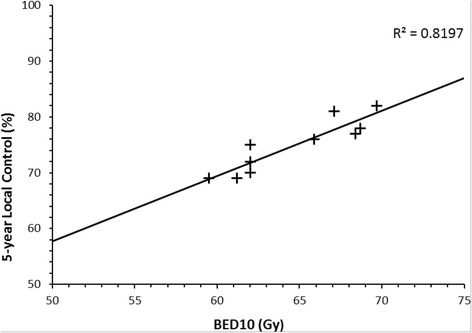
Relationship between BED10 and 5-year Local Control

These BED values are much more similar, in accordance with the similar local control rates for these regimens. Literature was reviewed to determine 5-year LC outcomes for T2 glottic cancers, where radiotherapy doses were quoted to allow BED to be calculated (Table 3). These BED values were plotted against local control rates to give the dose response graph (Fig. 1).

**Table 3:** Dose response series for local control and late effects

Series	Total Dose (Gy)	Dose per fraction (Gy)	OTT (days)	BED10^a^(Gy)	5-year LC (%)	BED3 (Gy)	Late effects (%)
Current series, 2017	52.5	3.28	22	69.7	82	110	1.8
Ermis, 2015 [10]	55	2.75	28	67.1	81	105	1.6
Motegi, 2015 [15]	64.8	2.4	37	68.4	77	117	4.5^b^
Berwouts, 2015 [16]	70	2	47	62.0	75	117	nr
Trotti, 2014 [17]	79.2	1.2	45	68.7	78	111	4.2
Trotti, 2014 [17]	70	2	47	62.0	70	117	2.5
Karasawa, 2013 [41]	63	2.25	41	61.2	69	110	0.0
Chera, 2010 [9]	65.3	2.25	39	65.9	76	114	1.2
Garden, 2003 [7]	70	2	47	62.0	72	117	2.6
Warde, 1998 [5]	50	2.5	28	59.5	69	92	nr

^a^Using *L* = 25, *T* = 1; ^b^For T2 subgroup; *nr* not reported

There are weaknesses in radiobiological modelling for tumour and normal tissue parameters using a linear quadratic formula including heterogeneity in patient, tumour and treatment characteristics. Even when homogeneous data are used to characterise tumour control (Table 3), assumptions are made for α/β ratio, duration of lag phase and time factor [11]. Accepting these weaknesses, the dose response gradient for T2 glottic cancer shows an absolute increase in local control of nearly 12%, with a 10Gy increase in BED. Previously reported data for T3 disease has shown an increase in local control of approximately 15% for a 10Gy increase in BED, albeit calculated using different time parameters [28].

### Control versus toxicity

The other consideration is that while improved local control is an attractive prospect, this should not be at the expense of increased serious late toxicities. However, shorter hypofractionated regimens for early glottic larynx cancer, which use small treatment volumes, have a low rate of severe late effects, approximately 1.1–1.8% [10, 15], which is comparable to a conventionally fractionated schedules of 70 Gy in 2 Gy/# (1.3–2.6%) [7, 18]. Studies of T1 glottic cancers using 3.125 Gy/# have shown no severe acute complications following treatment, which suggests hypofractionated regimes are safe and well tolerated [29, 30]. Dinshaw et al. reported persisting radiation oedema in 28% of patients with T2 glottic cancers treated with 2–3.3 Gy/#, however there was no correlation between toxicity and increasing fraction size [31]. Our toxicity data is limited by being retrospective, and only recording severe grade 4 toxicity requiring surgery, so a recommendation for further studies would be to record all grades of toxicity prospectively.

**Fig. 2:**
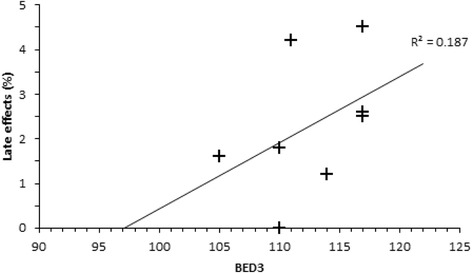
Relationship between BED3 and Late effects

Using further radiobiological modelling, where α/β = 3 for late responding normal tissues, the biologically effective dose (BED3) was calculated for both conventional and hypofractionated regimens, to compare the impact on these late effects (Table 3). BED3 was higher for the conventional dose fractionation schedule, and lower for hypofractionated treatments (Fig. 2), supporting the finding that late effects were not increased with these hypofractionated schedules.

### Chemoradiotherapy

As suggested by previous studies, T2b compared with T2a tumours had worse local control rates [4, 32]. We suggest the subdivision of T2 glottic tumours into T2a (normal vocal cord mobility) and T2b (impaired mobility) should be included in the TNM classification. Synchronous platinum based chemotherapy with radical radiotherapy is now standard of care for T3 glottic cancers and has been shown to improve outcomes in T2 disease [33–35]. A retrospective review by Bhateja et al. showed a trend towards inferior local control for T2b cancers treated with radiotherapy alone, compared to those with more advanced disease treated with chemoradiotherapy [33]. A limitation of this study was a lack of toxicity and quality of life data, which is important when balancing risks and benefits of intensifying treatment. Akimoto et al. investigated 63 patients with T2N0 disease, and found that chemoradiotherapy improved 5-year OS and 5-year disease-free survival compared to radiotherapy alone (96 and 89% vs. 87 and 68%, *p* < 0.05) [34]. In a multicentre review, Hirasawa et al. found an improvement in 5-year LC with chemoradiotherapy for T2 glottic cancers compared to those treated with radiotherapy alone (80.7% vs 64.4%, *p* = 0.149) [35]. Given the poorer outcomes associated with T2b disease, it may be worth considering concurrent chemotherapy in these patients, where the benefits may outweigh the potential increase in toxicity.

### Surgery

Systematic reviews of early glottic cancers have found similar outcomes between radiotherapy and surgery for patients with T2 glottic cancers [2, 36]. The average 5-year LC was 76% for radiotherapy compared to 77% for transoral laser microsurgery (TLM), however there were no randomised control trials identified for inclusion in this review. Similar outcomes for T2 glottic cancers treated by TLM were reported by Ansarin et al., with 75% 5-year disease-free survival [37]. Grant el al. achieved 93% 5-year LC for T2 glottic cancers after TLM, suggesting this is a safe and effective treatment option [38].

Voice quality following surgery is generally felt to be inferior to radiotherapy, however a review by Speilmann et al. found comparable vocal and quality of life outcomes for radiotherapy and TLM for early glottic cancers [39]. The ENT-UK Head and Neck Group recommended that TLM is offered to all patients with early glottic tumours up to T2a, as a standard of care [40]. Given the poorer outcomes for T2b glottic tumours, alternative management options including surgery should be considered for this subgroup of cancers.

## Conclusion

An accelerated hypofractionated radiotherapy schedule of 52.5 Gy in 16 once daily fractions for T2N0 glottic larynx cancer resulted in a 5-year local control rate of 82%. There was a low rate of severe late toxicity, which required surgical intervention.

Recent national guidelines acknowledge that for early glottic cancers, hypofractionated radiotherapy schedules have equivalent outcomes to conventional fractionation, without any increase in toxicity, and therefore suggest using regimes with fraction sizes between 2.65 and 3.25 Gy [20]. Our work supports this recommendation, and we suggest that for early glottic larynx cancers treated using small radiation volumes, hypofractionated radiation schedules are considered the standard of care.

## Abbreviations

BED: Biologically effective dose
CT: Computed tomography
d: Dose per fraction
D: Total dose
Gy: Gray
Gy/#: Gray per fraction
L: Time lag
LC: Local control
OS: Overall survival
OTT: Overall treatment time
T: Time factor
TLM: Transoral Laser Microsurgery
